# Visible Light Photocatalytic Degradation of Methylene Blue Dye and Pharmaceutical Wastes over Ternary NiO/Ag/TiO_2_ Heterojunction

**DOI:** 10.1021/acsomega.3c01766

**Published:** 2023-10-20

**Authors:** Widad Mohammed, Maha Matalkeh, Rola Mohammad Al Soubaihi, Ahmed Elzatahry, Khaled M. Saoud

**Affiliations:** † Material Science and Technology Program, College of Arts and Sciences, Qatar University, 2713 Doha, Qatar; ‡ Liberal Arts and Science, Virginia Commonwealth University School of Arts in Qatar, PO Box 8095, Doha, Qatar; § Functional NanoMaterials Group, Department of Applied Physics, School of Engineering Sciences, KTH Royal Institute of Technology, Hannes Alfvéns väg 12, 11419 Stockholm, Sweden

## Abstract

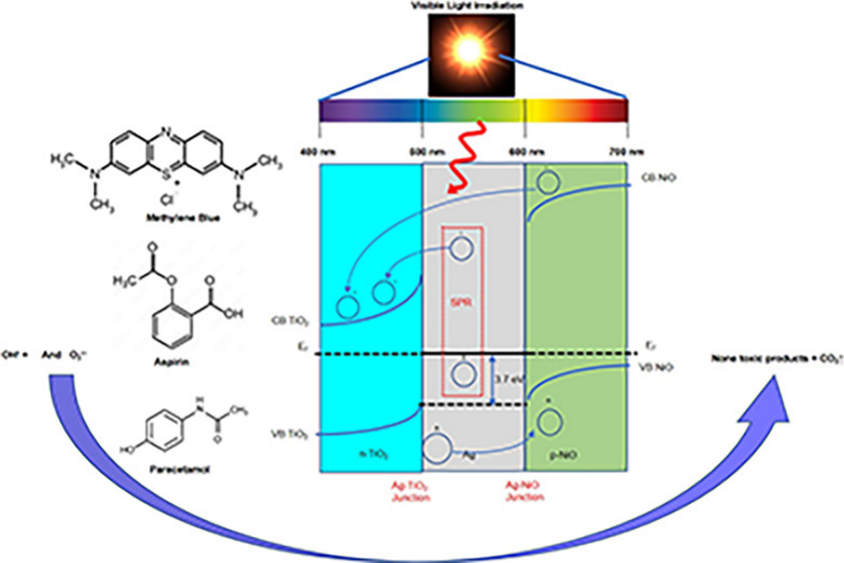

Ternary NiO/Ag/TiO_2_ heterojunction photocatalyst was prepared by deposition coprecipitation for visible light photocatalytic applications. Physicochemical properties of the synthesized NiO/Ag/TiO_2_ composite were characterized by X-ray diffraction, Brunauer–Emmett–Teller surface area measurement method, transmission electron microscopy, energy-dispersive X-ray spectroscopy techniques, X-ray photoelectron spectroscopy technique, and ultraviolet–visible absorption spectroscopy. The results suggest that the well-dispersed small metallic silver nanoparticles (<3 nm) facilitate electron transfer and bridge nickel oxide and titanium oxide. The photocatalytic degradation and the methylene blue (MB) dye kinetics were carried out on a ternary NiO/Ag/TiO_2_ composite and compared to bare TiO_2_ under visible light irradiation. The results indicate that NiO/Ag/TiO_2_ has superior MB photodegradation efficiency with a high reaction rate constant and low degradation time (93.15% within 60 min) compared to Ag/TiO_2_, NiO/TiO_2_, and bare TiO_2_. NiO/Ag/TiO_2_ nanocomposite was also investigated for the most common pharmaceutical waste degradation and exhibited excellent degradation efficiency. The enhancement of the composite’s performance could be attributed to the surface plasmonic resonance of the Ag nanoparticles, the formation of Schottky junctions at the Ag–TiO_2_ and Ag–NiO interface, and the p–n heterojunction between NiO and TiO_2_. Ag NPs act as a photosynthesizer and a photocatalyst, facilitate electron transfer, shift the absorption to the visible light region, reduce the band gap of TiO_2_, suppress the electron–hole recombination, and enhance the photocatalytic activity and stability as a result.

## 1 Introduction

Organic pollutants, such as phenolic chemicals, dyes, pigments, and microorganisms, have been found in water sources and on surfaces. More specifically, dyes such as methylene blue (MB) are considered one of the most harmful contaminants in surface and groundwater.^1−3^ Furthermore, pharmaceutical compounds such as aspirin (ASP) and paracetamol (PCM) are the most widely used drugs available to purchase over the counter without any prescription. These compounds contain acetylsalicylic acid and acetaminophen as active agents and aromatic ring groups, which affect the photodegradation of these compounds.^4^ Such pollutants are hard to remove using a traditional catalyst such as a commercial TiO_2_ catalyst.^2^ From the perspective of possible applications, it is critical to create successful light-triggered water purification materials.

Nanomaterials are characterized by their small size, high surface area, and excellent physical and chemical properties compared to bulk materials. Recent studies report the synthesis of effective nanomaterials for different applications in many fields, such as energy storage, electrodes in batteries or solar cells, or environmental applications such as CO_2_ reduction, water splitting, water waste treatment, photocatalysts, and disinfection agents.^5−9^

Photocatalysts have recently gained much attention due to their uses in environmental and health applications.^10^ For treating water and air pollution, photocatalysts synthesized from heterogeneous metal and metal oxide semiconductors^11^ are frequently used. Additionally, it is proven efficient in removing organic dye pollutants and pesticides when exposed to ultraviolet (UV) light.^12^ The catalyst’s capacity to generate electron–hole pairs is considered one of the crucial factors in determining the photocatalytic activity of the catalyst, which results in the production of free radicals that may cause further reactions.^13^ The most effective photocatalysis uses semiconducting nanoparticles to break down organic molecules, dangerous chemicals, bacteria, and viruses into CO_2_ and H_2_O. Metal-oxide nanoparticles, such as TiO_2_, ZnO, CuO, and WO_3,_ are known for their excellent photocatalytic properties and have been widely used to degrade pollutants and microorganisms under UV irradiation.^14,15^

In particular, nanostructured TiO_2_ is a well-known photocatalytic material with a long history of industrial applications. Using TiO_2_ photocatalyst under various irradiation conditions has been proven to remove contaminants and bacteria with excellent efficiencies.^1,16^ TiO_2_ has limitations such as wide optical band gap energy (3.2 eV), which requires intense UV irradiation and rapid recombination of photoexcited electron–hole that restricts its application in the visible light region of the solar spectrum and limits its efficiency for photocatalytic reactions despite its affordability, stability, and corrosion resistance.^17^ To improve the photocatalytic activity, shift the absorption in the visible light of the solar spectrum, and slow down the recombination of photoexcited electron–hole in TiO_2_, various strategies have been employed to change the electronic structure of TiO_2_, including doping, creating oxygen vacancies, or creating a junction with other photocatalytically active metal-oxide nanomaterials.^18^ Doping TiO_2_ with transition metals such as Ag, Ni, Cu, and Pd nanoparticles enhances the photocatalytic performance of TiO_2_.^19^ More precisely, Ag and Au have been widely utilized due to their ability to form Schottky heterostructures with TiO_2_. However, Ag nanoparticles are preferable due to the higher work function and lower cost than Au nanoparticles.^15,20−22^ Moreover, Ag nanoparticle doping leads to a higher electron injection efficiency into TiO_2_ owing to the surface plasmon resonance (SPR)-induced charge carriers of Ag nanoparticles.^23^ Hence, the photocatalytic performance of TiO_2_ will be enhanced due to the SPR excitations of Ag nanoparticles in the short-wavelength region of the visible light spectrum (∼380–450 nm).^24^

It has been reported that combining Ag with copper oxide, graphene, and graphitic carbon nitride can boost the photocatalytic performance of Ag/TiO_2_ catalysts by shifting the absorption further in the visible light region.^25^ Similar behavior is observed for metallic nickel (Ni)^9,26,27^ and nickel oxide (NiO) nanoparticles,^26^ which is commonly identified as metallaphotocatalysis that requires a photocatalyst to harness the energy of visible light and transfer it to the transition metal catalyst to initiate chemical reactions and photocatalytic degradation processes.^26^ Bare NiO is a cost-effective and less toxic photocatalyst with high activity, stability, and strong degradation efficiency toward organic contamination.^28^ Ag supported on NiO shows excellent photodegradation efficiency toward cationic (methyl violet) and anionic (methyl orange) dyes at room temperature.^29^ NiO is a p-type semiconductor with high mobility of charge carriers and is well-known to form an effective p-n heterojunction with n-type metal oxides such as TiO_2_ will, forming Ti^3+^/O_v_ in the Fermi level in the band gap of TiO_2,_ resulting in a substantial enhancement in the separation efficiency of charge carriers.^30^

Recent research articles focused on synthesizing highly active and green ternary nanocomposite photocatalysts for water contamination remediation and environmental application.^31^ AgNi alloy nanoparticles have attracted much attention as they are a new type of bimetallic alloy nanoparticles with a ratio of Ag_0.6_Ni_0.4_, expressing the best catalytic activity.^32−34^ Forming a ternary heterojunction by matching band positions between combining two semiconductors with a metal–semiconductor can facilitate charge transfer, drastically reduce the electron–hole recombination, and increase light absorption in the visible light region of the electromagnetic spectrum and hence significantly augment the catalytic efficiency.^8,35^ Supporting AgNi alloy nanoparticles on metal oxides such as ZnO and MoS_2_ to form ternary photocatalysts showed excellent catalytic activity toward contaminations and organic dyes.^33,36^

To our knowledge, a few papers in the literature report the synthesis and photocatalytic activity of a ternary NiO/Ag/TiO_2_ heterojunction photocatalyst for water splitting and organic MB dye and azo dye (methyl red) degradation. NiO/Ag/TiO_2_ composite was synthesized using various methods, including the irradiation method,^37^ sonication-based wet chemical synthesis,^38^ and gas phase using a spark discharge generator.^39^

This study reports the synthesis and characterization of ternary NiO/Ag/TiO_2_ heterojunction nanocomposites, which involve the formation of ternary Schottky heterostructures between (NiO–TiO_2_), (Ag–NiO), and (Ag–TiO_2_) for photocatalytic pollutant degradation under visible light irradiation_._ The photocatalyst combines the SPR-induced charge carriers of Ag nanoparticles with the high mobility of charge carriers NiO and the formation of effective p-n heterojunction with TiO_2_ where the Ag nanoparticles bridge between NiO and TiO_2_, resulting in a dramatic drop in the recombination rate of the electron–hole leading to a substantial boost of its visible light photocatalytic activities. The NiO/Ag/TiO_2_ nanocomposite with 1% Ag and Ni of ratio loading (0.6:0.4) was synthesized using a novel deposition coprecipitation wet chemical method. The photocatalytic activity of the NiO/Ag/TiO_2_ nanoparticles was studied toward the visible light degradation of MB and pharmaceutical waste such as ASP and PCM, which was found to be significantly high. Moreover, the nanomaterial’s kinetic properties, MB degradation mechanism, effect of annealing, and recyclability were investigated.

## 2 Experimental Section

### 2.1 Materials

Titania (Nano TiO_2_; Rutile, Sigma-Aldrich), silver nitrate (AgNO_3_, HIMEDIA, India), nickel II nitrate 6-water [Ni (NO_3_)_2_. 6H_2_O, Breckland Scientific, UK], sodium borohydride (NaBH_4_, Fisher Scientific), ethanol (EtOH, Fisher Chemicals), ultrapure water (18.1 MΩ-cm, Thermo Scientific), MB (Sigma-Aldrich, USA), ASP (Bayer, USA), PCM (Pharma, USA), methanol (Honeywell, Germany), acetic acid (EMSURE, Germany), and hydrochloric acid (Scharlau, Spain) were all used without any further purification.

### 2.2 Catalyst Synthesis

The ternary NiO/Ag/TiO_2_ heterojunction nanocomposites were synthesized using coprecipitation deposition. Typically, 4 g of TiO_2_ (rutile) nanopowder was ultrasonically dispersed for 15 min in a 250 mL beaker in a mixed solution of 150 mL of ultrapure (UP) water and absolute ethanol in a volume ratio of 1:1. A ratio of 0.6:0.4 of Ag and Ni compositions was used. 0.0377 g of Ag NO_3_ and 0.0793 g of Ni (NO_3_)_2_ in 20 mL of a mixed solution of UP water and absolute ethanol in a volume ratio of 1:1 were separately mixed for 15 min. The mixture was then added to the TiO_2_ solution and stirred for 15 min. About 8 mL of 0.1 M NaBH_4_ was added to the solution using a titration method until the solution color changed from white to light brown, and then the solution was stirred for 1 h. The precipitated nanopowder was separated by centrifugation, followed by washing three times with UP water and absolute ethanol and dried at 60 °C for 24 h. For comparison, 1% Ag/TiO_2_ and 1% NiO/TiO_2_ nanocatalysts were synthesized using the same conditions as the previous steps. Schematic of the synthesis procedure is shown in Figure S1 in the supplementary file.

### 2.3 Catalyst Characterization

N_2_ sorption analysis was performed at 77 K using the Micromeritics ASAP2020 accelerated surface area and porosity system (Micrometrics, Norcross, GA, USA), with specific surface area and pore size distribution measured using Brunauer–Emmett–Teller (BET) and Barrett–Joyner–Halenda (BJH) methods, respectively. Catalyst structure, crystallography, and phase analysis were performed using X-ray diffraction (XRD) (PAN Analytical Empyrean) with Cu–Kα (λ = 0.15418 nm) in the range of 10° to 99.9°. Transmission electron microscopy (TEM) was performed using the FEI model TECNAI G2, TF20 microscope. High-resolution transmission electron microscopy (HRTEM) (FEI Talos 200X) was used at an operating voltage of 200 kV, and energy-dispersive X-ray spectroscopy (EDS) (Thermofisher Scientific, USA) was used to analyze the morphology and elemental mapping of the catalyst produced. X-ray photoelectron spectroscopy (XPS) was used to measure the concentration, chemical state, and surface condition of silver nanoparticles, and Casa XPS software was used for data analysis and peak fitting. The obtained XPS spectra were calibrated to the C 1s feature at 284.6 eV.

UV–vis absorption spectra of the prepared samples were acquired using a split-beam UV–vis spectrophotometer (YK Scientific, UV1810/UV1810S, China).

### 2.4 Photocatalytic Activity Test

The catalyst’s efficiency was evaluated using a MB dye in an aqueous solution. A 400 W halogen lamp (metal halide) (λ ≥ 400 nm, with λ_max_= 546 nm) with UV cut filter coating was used in all photodegradation experiments. The intensity of the light source was ∼170 W/m^2^ measured by a light meter (EXTECH Instrument, Model 401027, Luximetro de Bolsillo) at a distance of 15 cm between the lamp and the cup using different doses of photocatalyst (0.02, 0.04, and 0.08 g) suspended in 80 mL of MB (5.0 mg/L) solution in a 150 mL beaker. The solution was kept under continuous stirring in the dark for 30 min before irradiation to balance the absorption and desorption processes between the produced photocatalyst and the MB solution. The maximum absorption wavelength of the MB dye was measured using the UV–visible spectrum at 664 nm. Absorbances were recorded at 10 min intervals for 60 min to determine the MB dye’s deterioration. In the control experiment, the aqueous solution was irradiated without a photocatalyst. In all tests, the blank sample consisted primarily of water. UV–vis spectrometry measured the MB concentration after removing the catalyst from the liquid using a 0.45 μm PTFE syringe filter.

The photocatalytic kinetics of MB degradation was described by the pseudo-first-order kinetic model for dye degradation efficiency as follows:
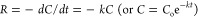
1where *R* represents the reaction rate, *C*_o_ and *C* (g/L) are the initial reactant concentration of aqueous MB solution and the concentration after time *t*, respectively, *k* is the reaction rate constant, and *t* is reaction time according to the pseudo-first-order kinetics.^15^

Integrating eq 1, the kinetic model can be expressed by the following equation:
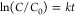
2

The reaction constant k can be calculated from the plot of ln(*C*/*C*_0_) versus time, yielding a linear plot with the slope *k*. Furthermore, the degradation efficiency can be calculated using eq 3:
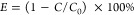
3

### 2.5 Pharmaceutical Waste Degradation Test by Using a UV–Vis Spectrophotometer

Photocatalytic degradation of pharmaceutical compounds was performed by using the same experimental setup and conditions used for the MB photodegradation test. ASP and PCM were applied to test the degradation efficiency of the nanocatalyst for pharmaceutical waste decomposition. The concentration of each pharmaceutical compound was 2 ppm, along with a catalyst dose of 1 g/L in solution. The maximum absorption wavelength was measured at 197 nm by using the UV–visible spectrum. Photocatalytic particles were separated from the solution by using a 0.45 μm Millipore filter.

### 2.6 Pharmaceutical Waste Degradation Test by Using High-Pressure Liquid Chromatography

A C18 reversed-phase column was used to identify the photodegradation of PCM and ASP. However, PCM, ASP, and their byproducts were enhanced by employing a mobile phase of methanol and acetic acid in 1% water with a v/v ratio 40/60. The mobile phase was prepared fresh and filtered with a 0.22 μm membrane and sonicated for 5 min. The device was operated at 25 °C with a flow rate of 1 mL min^–1^. Moreover, the samples were injected with a volume of 20 μL and detected at 243 and 276 nm for PCM and ASP, respectively. A fresh aqueous stock solution was prepared separately with a concentration of 1 g/L of each component (PCM and ASP) to prepare the tested samples. Then, both the compounds were diluted to reach the concentration of 2 ppm in acidic water that was adjusted by adding HCl to reach pH = 3. After that, 1 g/L catalyst was added to each medicinal solution. The same visible light simulation used in all experiments was applied for 120 min. The tested samples were taken to analyze using high-performance liquid chromatography (HPLC) at 0, 40, 80, and 120 min after being filtered by a 0.45 μm filter membrane to separate the nanoparticles.

## 3 Results and Discussion

### 3.1 Catalyst Characterization

The surface area and porosity of the nanocomposite were measured quantitatively using nitrogen adsorption BET. The samples were degassed at 150 °C for 48 h before recording N_2_ adsorption–desorption isotherms at 77 K (−196 °C). The BET surface area and porosity (i.e., pore volume and pore size) of NiO/Ag/TiO_2_ and TiO_2_ are listed in Table 1. Noticeably, the surface area, pore volume, and pore size of NiO/Ag/TiO_2_ nanocomposites increased compared to rutile TiO_2_.^40^ It is significant to mention that the photocatalytic performance is influenced by the surface area and pore structure parameters (size and volume); the higher the pore volume, porosity, and surface area, the higher the possibility for the catalyst to interact with and adsorb the pollutant.^41^

**Table 1:** Summary of BET-Specific Surface Area, Pore Volume, and Pore Size of the Synthesized Sample of Ternary Titania-Supported Silver–Nickel Oxide Nanocomposite (NiO/Ag/TiO_2_) and Bare Titania (Rutile TiO_2_)

material	BET-specific surface area (m^2^ g^–1^)	pore volume (cm^3^/g)	pore size (A)
TiO_2_	12.967	0.0194	30
NiO/Ag/TiO_2_	14.315	0.0266	37.2

**Figure 1:**
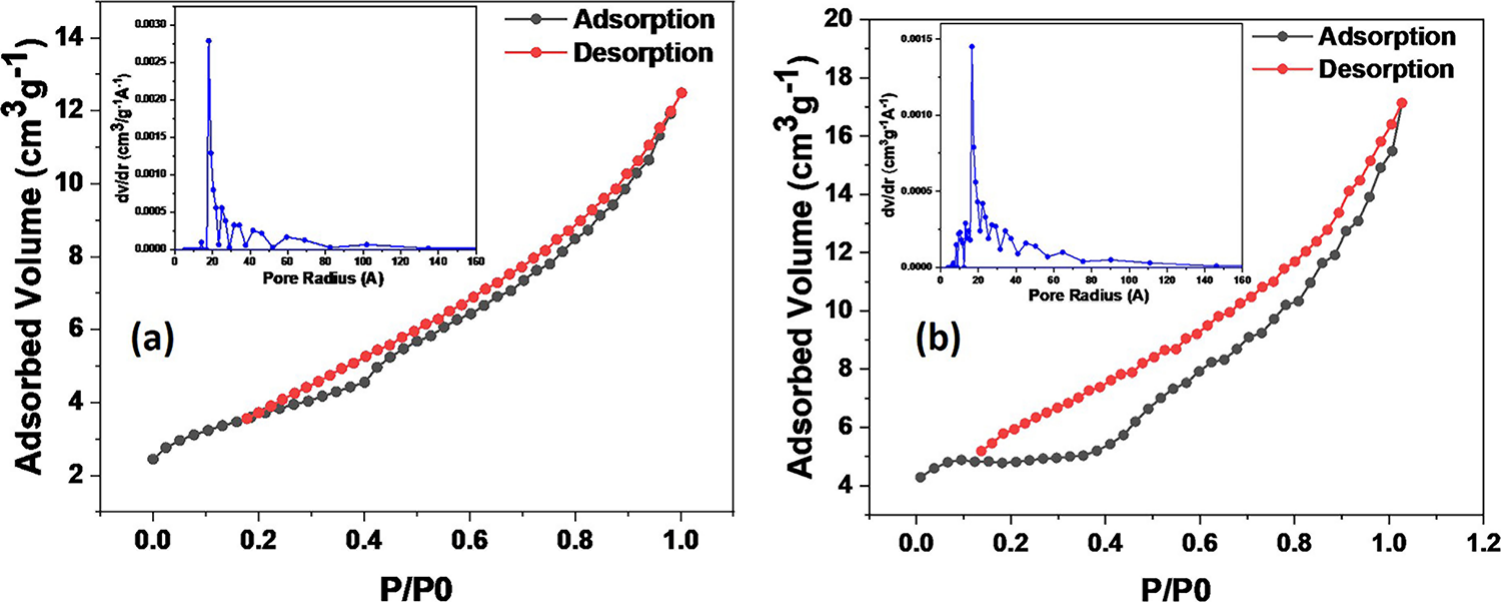
N_2_ adsorption–desorption isotherms and the corresponding BJH adsorption–desorption pore volume distribution of (a) TiO_2_ rutile and (b) NiO/Ag/TiO_2_ nanocomposites.

Figure 1 shows the N_2_ adsorption–desorption isotherms of ternary NiO/Ag/TiO_2_ nanocomposites and bare TiO_2_ (rutile). The specific surface area, porosity, pore volume, and pore size distribution of NiO/Ag/TiO_2_ nanocomposites were done using a Rise 1010 surface area and porosity analyzer (Jinan Rise Science and Technology Co., ltd., China) by N_2_ physisorption.

**Figure 2:**
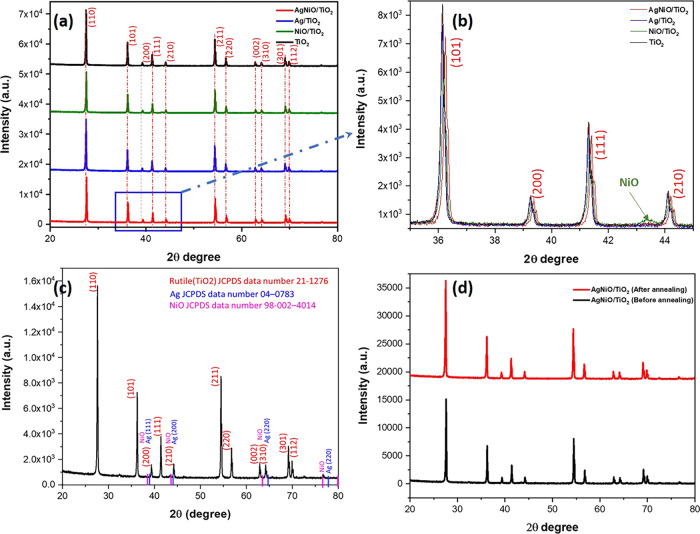
XRD patterns of (a,b) comparison of the four composites (NiO/Ag/TiO_2_, TiO_2_, Ag/TiO_2_, and NiO/TiO_2_), (c) XRD of NiO/Ag/TiO_2_ with corresponding cards for rutile TiO_2_, Ag, and NiO, (d) NiO/Ag/TiO_2_ before and after annealing.

The XRD patterns of the fresh samples show the rutile TiO_2_ (JCPDS data number 21-1276).^42^Figure 2a,b demonstrates an overlay of XRD diffraction peaks of the NiO/Ag/TiO_2_, TiO_2_, Ag/TiO_2_, and NiO/TiO_2_ nanocomposites. Figure 2c shows the XRD diffraction pattern of NiO/Ag/TiO_2;_ the observed peaks are assigned to the tetragonal rutile TiO_2_ phase only, with no other phase of TiO_2,_ Ag (JCPDS data number 04-0783),^43^ or NiO (JCPDS data number 98-002-4014).^44^ A careful comparison of the XRD patterns for NiO/Ag/TiO_2_, TiO_2_, Ag/TiO_2_, and NiO/TiO_2_ nanocomposites showed a rightward shift in the peaks of NiO/Ag/TiO_2_ (Figure 2a,b). This rightward shift indicates a slight contraction in the lattice structure.^45^ A small peak is observed at 2θ= 34°, corresponding to the NiO peak in the XRD pattern of NiO/TiO_2_ nanocomposites. This peak was not observed in the XRD pattern of NiO/Ag/TiO_2_, suggesting that Ag plays a vital role in bridging the NiO and TiO_2_ nanoparticles. As indicated in Figure 2c, none of the Ag, NiO, AgO, or Ni peaks are observed due to the low content of Ag and Ni nanoparticles (∼1% loading) and the excellent dispersion within TiO_2_; also, the small size of the particles (<5 nm) cannot be detected in the XRD instrument.^37^ The highest reported Ag peaks for Ag nanoparticles appear at concentrations well above 8.1% Ag loading in the Ag/TiMoNO composite.^46^ These results agree well with the previously reported XRD pattern of NiO/Ag/TiO_2_.^37^ As shown in Figure 2d, there are no Ag and NiO peaks observed, and there is no significant difference between the XRD pattern of NiO/Ag/TiO_2_ before and after annealing, except the notable increase in the intensity and sharpness of the peaks, suggesting a more crystalline nature of the composites after annealing.

### 3.2 Analysis of the UV–Visible Spectra

**Figure 3:**
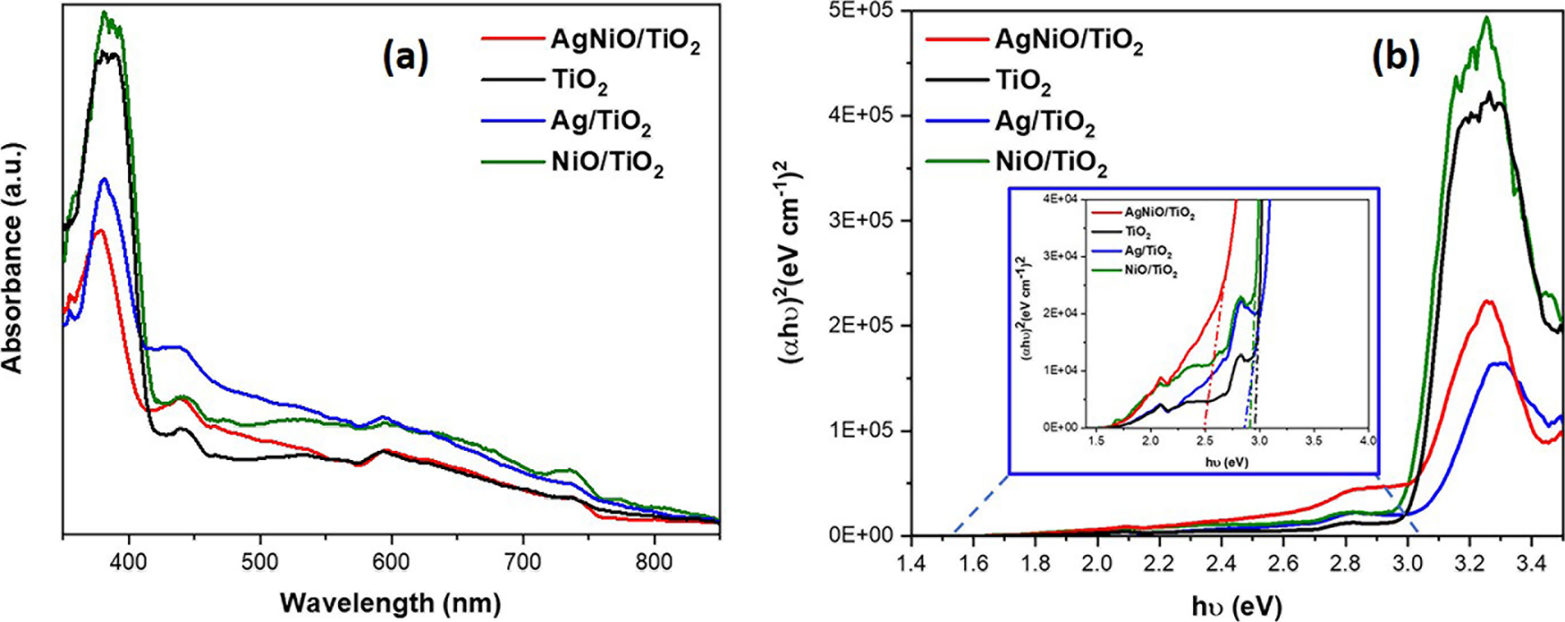
UV–vis spectra results: (a) Comparison of ternary titania-supported silver–nickel oxide nanocomposite (NiO/Ag/TiO_2_), titania (TiO_2_), titania-supported silver (Ag/TiO_2_), and titania-supported nickel (NiO/TiO_2_) and (b) band gaps of the composites using the Tauc plot of the corresponding results.

The optical properties and energy gap of the NiO/Ag/TiO_2_ photocatalyst were analyzed using UV–visible (UV–vis) spectroscopy and compared to those of bare TiO_2_ (rutile) as well as Ag/TiO_2_ and NiO/TiO_2_ composites, as shown in Figure 3. Figure 3a illustrates the optical absorbance of the samples within the range (350–900 nm). From Figure 3a, we can conclude that incorporating NiO and Ag nanoparticles into TiO_2_ significantly improved the composite’s absorption, evident by the redshift at 600 nm. Hence, the redshift of the peak to a longer wavelength will cause a reduction in the energy gap of NiO/Ag/TiO_2_ because of localized surface plasmon resonance (LSPR) being affected by the Ag nanoparticles.^6,22^ Besides that, the change of color to brown after synthesizing the NiO/Ag/TiO_2_ compared to the rutile TiO_2_ (white) is another evidence of optimizing the optical properties, which foster the visible light absorption (Scheme S1). Figure 3b shows the Tauc plot for all bare TiO_2_ alongside the NiO/Ag/TiO_2_, Ag/TiO_2,_ and NiO/TiO_2_ composites; the plot indicates that all composites have a direct band gap with band gap energies for NiO/Ag/TiO_2_, TiO_2_, Ag/TiO_2_, and NiO/TiO_2_ nanoparticles 2.50, 3.0, 2.85, and 2.92 eV, respectively, calculated using the Tauc plot method, as shown in Figure 3b. These results indicate that incorporating Ag and NiO into TiO_2_ reduced the band gap by 0.5 eV.

**Figure 4:**
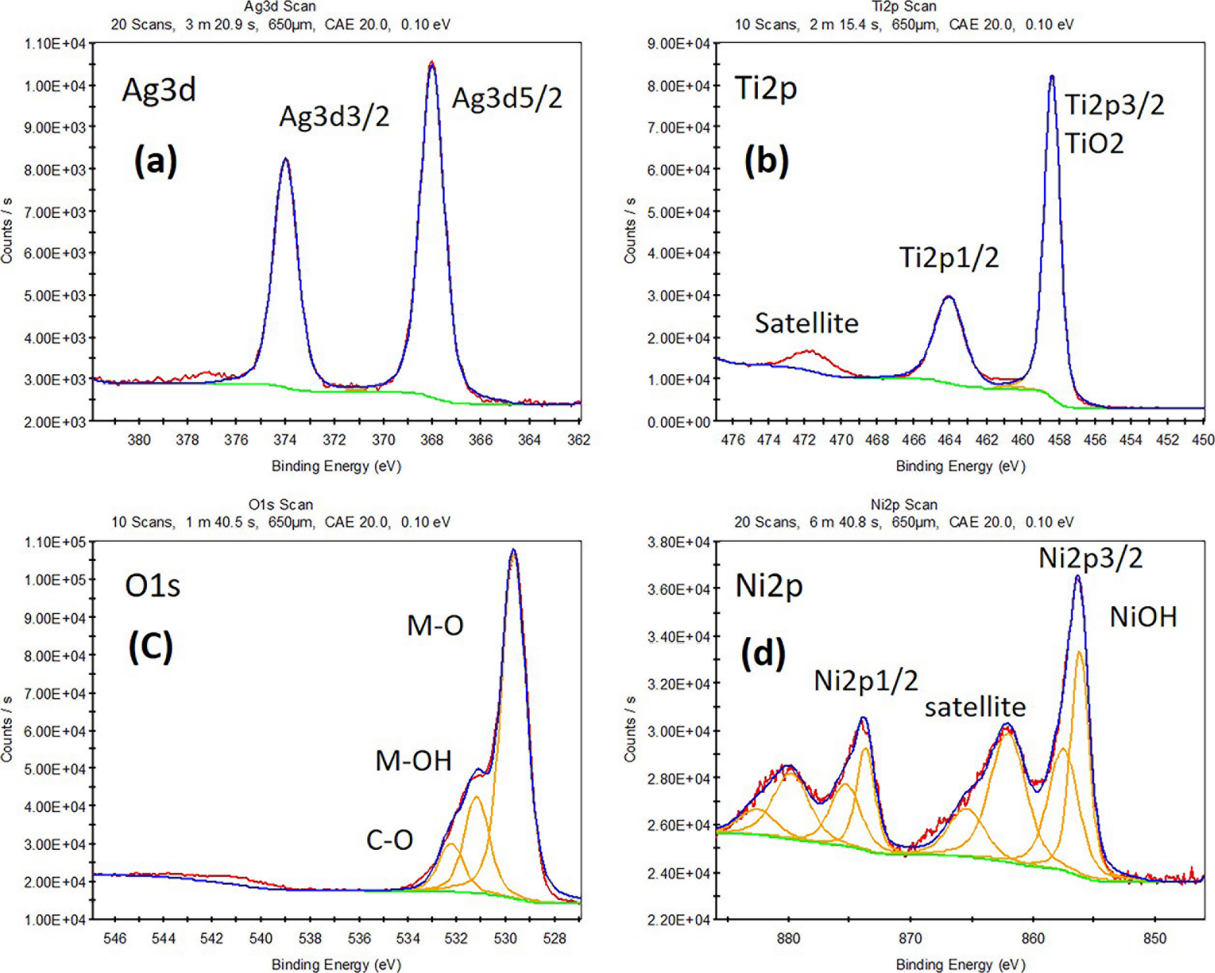
XPS analysis of ternary titania-supported silver–nickel oxide nanocomposite (NiO/Ag/TiO_2_): high-resolution spectra of (a) Ag 3d, (b) Ti 3d, (c) O 1s, and (d) Ni 2p.

XPS analysis of the NiO/Ag/TiO_2_ nanocomposite was performed, as represented in Figure 4. The survey scan for the chemical components of the composite is indicated in Figure S1. XPS peaks show little contamination in the sample containing Ni 2p, Ti 2p, Ag 3d, and C 1s. According to Figure 4a–d, there were two peaks in the Ti 2p binding energy region. Ti 2p_1/2_ is attributed to the peak at 464.7 eV, whereas Ti 2p_3/2_ is attributed to the peak at 459.1 eV. The 5.6 eV split between Ti 2p_1/2_ and Ti 2p_3/2_ core levels confirms that Ti^4+^ is the stochiometric phase of TiO_2_. The large doublet peaks centered at binding energies of 459.1 and 464.7 eV correspond to Ti^4+^ 2p_3/2_ and Ti^4+^ 2p_1/2_, respectively, and the small doublets at 458.7 and 464.5 eV correspond to Ti^3+^ 2p_3/2_ and Ti^3+^ 2p_1/2_ binding energies, respectively. A small amount of Ti3+ species formed compared to the pure TiO_2_ sample can be attributed to the doping of Ni^3+^ and Ag^+^ ions in TiO_2_.^39^

O 1s scan showed overlapping peaks of O^2–^ which combined Ti^4+^ and small Ti^3+^ species at the peak of 530 eV, and the other peak ascribes to the Ni–O bonding at the peak of 531. In addition, the peak of 531.5 reveals surface-adsorbed oxygen species such as the −OH group or chemisorbed H_2_O and oxygen vacancies, and the peak of 532.2 could be attributed to C–O bonding or which comes from the XPS instrument. The Ni 2p scan peaks that appeared at 873.2 and 855.6 eV represent the forming of the Ni–O bond, ascribed to the Ni^2+^ 2p_1/2_ and Ni^2+^ 2p_3/2_ signals, respectively. No peak indicated the presence of metallic Ni (852.7 eV), which showed that the Ni completely oxidized on the surface.^47^ The other labeled peaks in the spectrum are related to the satellite peaks of 2p_3/2_ and 2p_1/2_. The XPS spectrum also suggests that the chemical state is mainly Ni^2+^ with the absence of Ni^3+^ chemical states on the surface of NiO/Ag/TiO_2_. The Ag 3d scan shows a deconvolution of Ag 3d_5/2_ and Ag 3d_3/2_ spectra at binding energies of 368.3 and 374.3 eV, respectively, with the splitting of the 3d doublet of Ag of 6.0 eV, revealing the presence of Ag^0^ (metallic Ag) as reported in previous work.^48,49^ Furthermore, the NiO/Ag/TiO_2_ sample is shifted toward higher binding energies than the pure TiO_2_ sample, which can be attributed to the electronic interactions between p-NiO and n-TiO_2_ nanoparticles.^39^

**Figure 5:**
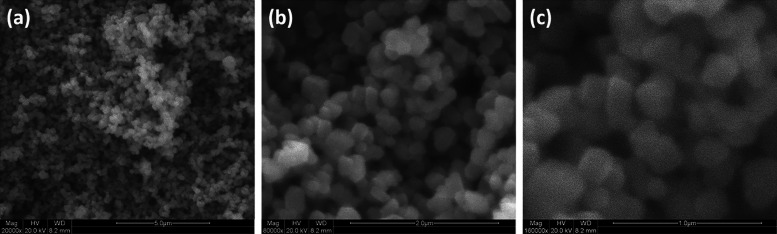
(a–c) SEM images of NiO/Ag/TiO_2_ nanoparticles at different magnifications (5, 2, and 1 μm, respectively).

We performed SEM analysis on the synthesized sample to investigate the morphology of the NiO/Ag/TiO2 nanoparticles. The morphology and structure of the NiO/Ag/TiO_2_ nanoparticles are displayed in Figure 5a–c. As seen from the SEM images, the TiO_2_ nanoparticles have an irregular spherical shape with an average size of 200 nm. Also, the micrograph showed that the small TiO_2_ nanoparticles undergo agglomeration to form large TiO_2_ particles, as observed in Figure 5 a–c.

**Figure 6:**
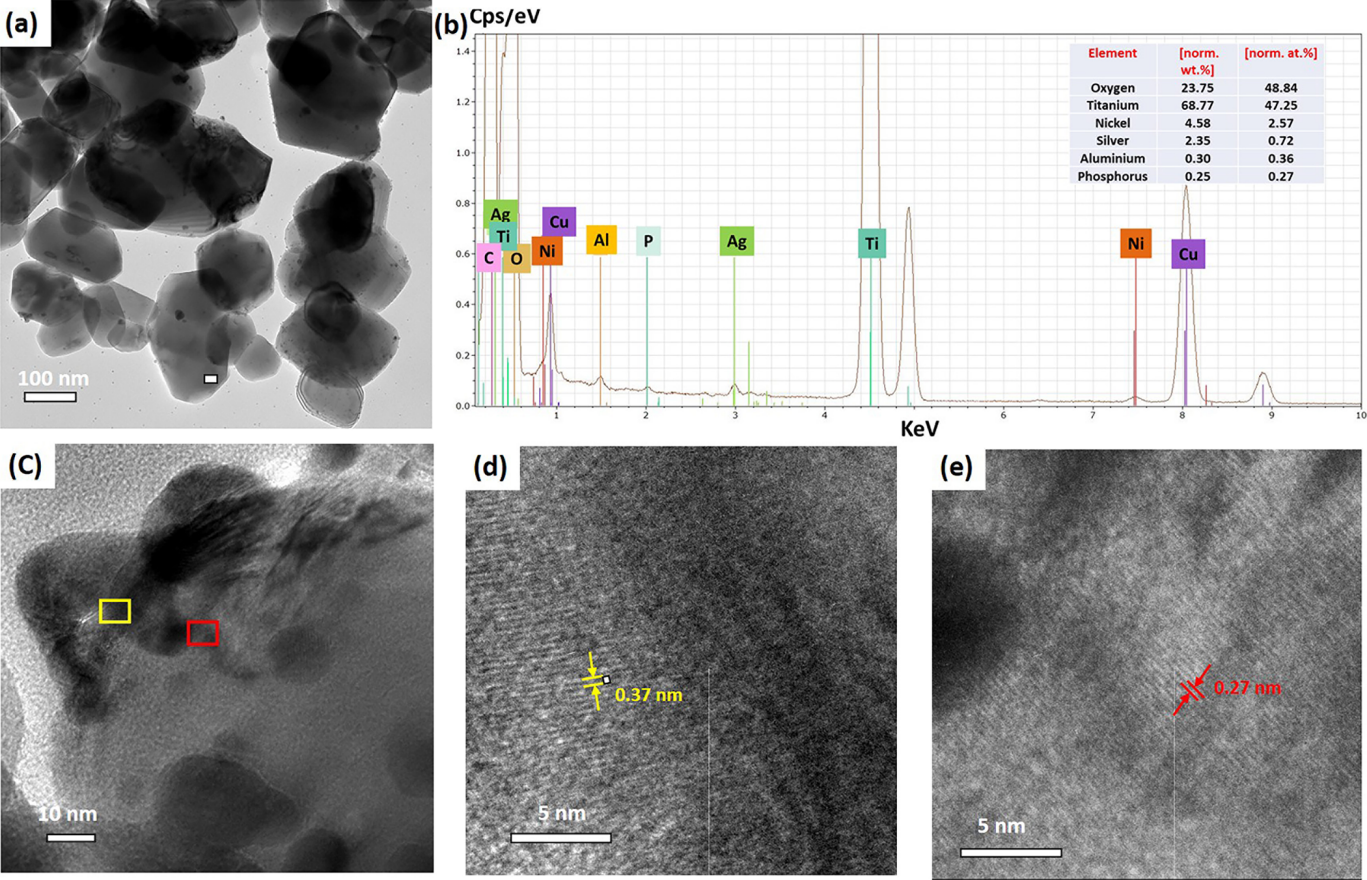
(a) HRTEM image, (b) EDS elemental analysis of ternary titania-supported silver–nickel nanocomposite (NiO/Ag/TiO_2_) composite, and (c–e) TEM images of NiO/Ag/TiO_2_ at different magnifications (10 and 5 nm).

The HRTEM image of the NiO/Ag/TiO_2_ nanocomposite is shown in Figure 6. The micrograph showed a regular spherical shape and size of TiO_2_ with a size of about 150–200 nm; neither Ag nor NiO nanoparticles were observed in Figure 6a,c.^50^ According to the HRTEM images in Figure 6a,c, no Ag or NiO particles were observed. TEM-EDS elemental analysis was employed to determine the chemical composition, size, and distribution of the NiO/Ag/TiO_2_ nanoparticles. Figure 6b shows the quantitative EDS elemental analysis of Ag, Ni, and TiO_2_. The EDS of Ag, Ni, Ti, and O demonstrates that these three elements are well interdispersed and possibly exhibit a strong surface interaction. It is important to note that the EDS spectra contains trace amounts of aluminum and phosphorus which were not included in the synthesis and may be caused by cross-contamination or sample handling. EDS confirms the presence of Ag and NiO, which suggests that the Ag and NiO nanoparticles are mainly tiny and well-dispersed (<3 nm), revealing the interfacial contact between the Ag and NiO particles on the TiO_2_ surface.^51^

Furthermore, HRTEM image in Figure 6d,e shows that the lattice spacing of TiO_2_ rutile is 0.37 nm for the crystal plane [110] and 0.27 nm for the crystal plane [101], which confirms that the large particles are ascribed to the rutile TiO_2_.^32,33,36,37^

### 3.3 MB Dye Photodegradation over NiO/Ag/TiO_2_ Nanocomposites

#### 3.3.1 Photodegradation over NiO/Ag/TiO_2_, Ag/TiO_2_, and NiO/TiO_2_ Nanocomposites

**Figure 7:**
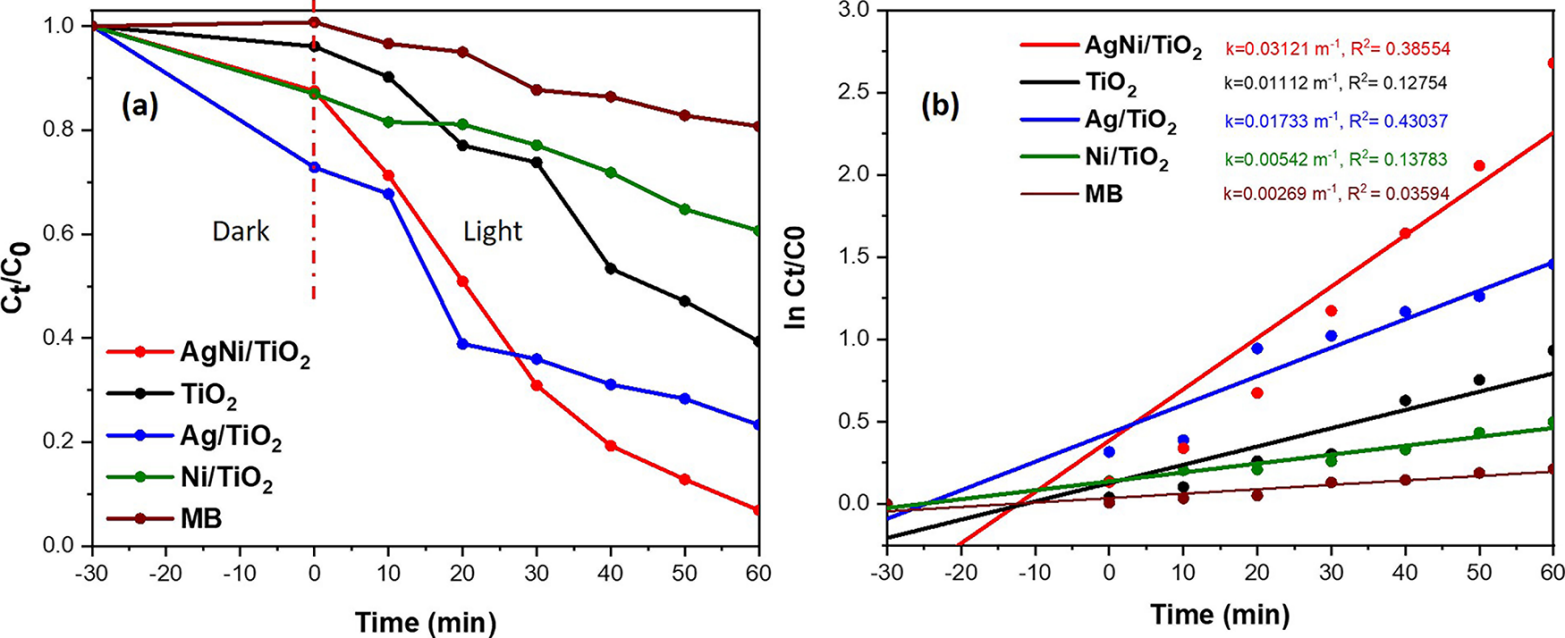
Photocatalytic degradation of MB solution. (a) Comparison of ternary titania-supported silver–nickel oxide nanocomposite (NiO/Ag/TiO_2_) with titania (TiO_2_) and other composites under visible light for 60 min. (b) Pseudo-first-order kinetics of the degradation of MB solution.

Photocatalytic degradation of MB solution by the NiO/Ag/TiO_2_ nanocomposite and other compounds was evaluated under visible light irradiation. NiO/Ag/TiO_2,_ 1% NiO/TiO_2_, 1% Ag/TiO_2,_ and bare TiO_2_ were tested as photocatalysts in aqueous MB dye (1 g/L) for 60 min. Absorbance was measured as a function of time by using C_t_/C_0_ versus time (minutes), as shown in Figure 7a. The photocatalytic degradation rate of MB of the composites in the dark (prior to the light irradiance) was tested and showed different degradation rate trends compared to the rates in the presence of light irradiance situation. The photodegradation rates were different due to the variation in the surface adsorption capacity in each material.^52^ Many factors affect the catalyst surface adsorption, such as the BET surface area, pore size, and pore volume (Table 1).^52^ This can explain why NiO/TiO_2_ and NiO/Ag/TiO_2_ have higher surface absorption than TiO_2_. Also, the higher amount of small particles of Ag (1%) on the surface of Ag/TiO_2_ might have the largest surface adsorption capacity in dark condition. NiO/Ag/TiO_2_ showed the highest photocatalytic reactivity among the tested compounds at 60 min; C_t_/C_0_ is 0.068 after visible light irradiation. Additionally, ln *C*_*t*_/*C*_0_ was plotted against the reaction time, as shown in Figure 7b, for all of the tested nanocomposites and MB with no catalyst. The slopes for this plot gave the reaction rate (k) by employing the fit linear equation regarding pseudo-first-order kinetics law. Table 2 summarizes each nanocatalyst reaction rate constant and degradation efficiency. Among all of the tested compounds, NiO/Ag/TiO_2_ shows the fastest reaction rate (0.03121 min^–1^) and the highest degradation efficiency (93.15%). This comparison leads to the finding that the novel nanocomposite catalyst (NiO/Ag/TiO_2_) has the best photodegradation rate for MB aqueous solution compared to other composites on TiO_2_ and bare TiO_2_. Furthermore, previous research reports that G-P25, G-TiO_2_, G-ZnO, and G-ZnO-CTAB reach 100% photodegradation of the MB aqueous solution in 80, 110, 90, and 150 min, respectively, under the same conditions.^53^ Also, a recent study identifies that 4% PVA-loaded ZnO-F photocatalyst achieves a high ability in the degradation of MB dye under visible light irradiation within 120 min.^7^ According to the fit linear equation, NiO/Ag/TiO_2_ can decompose MB solution completely in about 63 min. Therefore, NiO/Ag/TiO_2_ shows a much higher photocatalytic activity of the MB aqueous solution than the previous materials. In addition, recent work has demonstrated that 1% Ag/WO_3_ photocatalyst degrades 76.6% of the MB aqueous solution within 60 min under the same conditions.^20^ Consequently, NiO/Ag/TiO_2_ shows the best MB aqueous solution degradation efficiency. Moreover, previous investigation of decolorization of the MB aqueous solution by Au-doped TiO_2_-supported SWCNT hybrid nanocomposites found a compatible photocatalytic performance to the NiO/Ag/TiO_2_ nanocomposite.^6^

**Table 2:** Reaction Rate Constants and Degradation Efficiencies for Bare Titania (TiO_2_), 1% Ternary Titania-Supported Silver–Nickel Nanocomposite (NiO/Ag/TiO_2_), Titania-Supported Silver (Ag/TiO_2_), Titania-Supported Nickel (NiO/TiO_2_), and MB without Catalyst

material	reaction rate constant (min^–1^)	degradation efficiency (%)
NiO/Ag/TiO_2_	0.03121	93.15
NiO/TiO_2_	0.00542	39.4
Ag/TiO_2_	0.01733	76.73
bare TiO_2_	0.01112	60.7
no-catalyst	0.00269	19.3

#### 3.3.2 Kinetics Study of Photocatalytic Performance as a Function of Photocatalyst Dose

**Figure 8:**
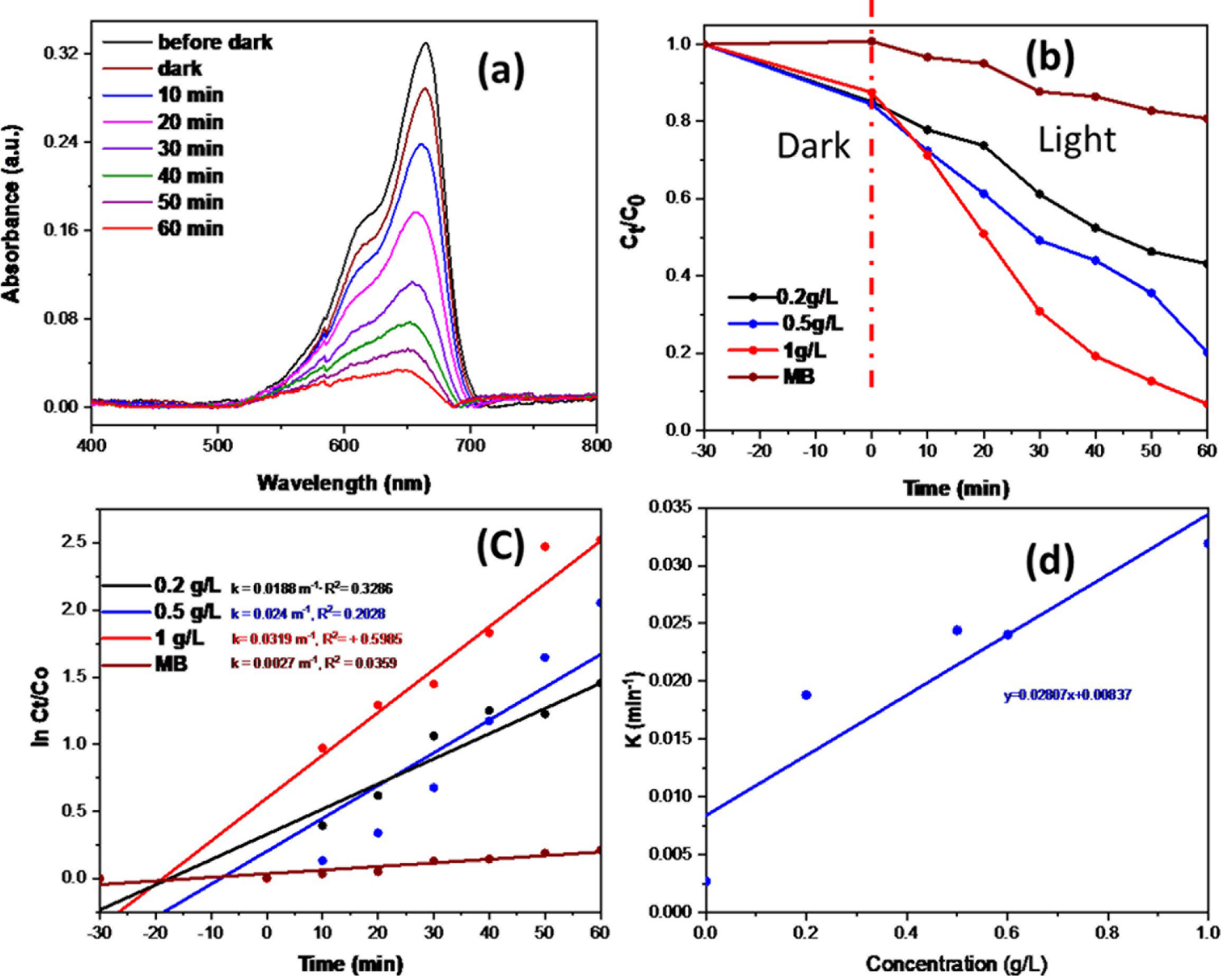
(a) UV–vis absorption spectra of MB solution with different degradation times with 1% of ternary NiO/Ag/TiO_2_ nanocomposite under visible light irradiation, (b) photodegradation of MB solution by different concentrations of NiO/Ag/TiO_2_ nanocomposite within 60 min under visible light irradiation, (c) pseudo-first-order kinetics for the degradation of NiO/Ag/TiO_2_ (0–1 g/L), and (d) apparent degradation rate on the catalyst concentration.

The catalyst dose significantly impacts the photodegradation efficiency of the contaminant. The degradation mechanisms that require less catalyst are preferable. This work used 0, 0.2, 0.5, and 1 g/L doses of the NiO/Ag/TiO_2_ nanocomposite photocatalyst for the photodegradation activity test of MB aqueous solution under visible light for 60 min. The absorbance vs wavelength plot, shown in Figure 8a, measures the amount of degradation of MB in the presence of 1g/L NiO/Ag/TiO_2_. The performance of the (0–1 g/L) doses of the NiO/Ag/TiO_2_ nanocomposite relative to C_t_/C_0_ versus time is demonstrated in Figure 8b. It shows that the MB solution’s photodegradation activity increases as the catalyst’s dose increases. The corresponding kinetic data of photocatalytic degradation of MB dye solution by the various doses of the NiO/Ag/TiO_2_ nanocomposite catalyst were obtained using the pseudo-first-order kinetic equation as ln *C*_t_/C_0_ versus time (min) (Figure 8c) Table 3 summarizes the reaction rate constants and degradation efficiencies for 1% NiO/Ag/TiO_2_ photocatalyst with different doses (0, 0.2, 0.5, and 1 g/L) in MB aqueous solution under visible light for 60 min. The 1 g/L dose of the catalyst has the highest effect on the MB solution’s photodegradation with a constant reaction rate reaching 0.0319 min^–1^, which is 1.7 times faster than the 0.2 g/L doses of the catalyst and 11.8 times faster than the MB solution without the catalyst. Thus, the catalyst is considered dose-dependent. In addition, Figure 8d demonstrates the rate constant of the photodegradation of the MB solution versus the concentration of (0–1) g/L of NiO/Ag/TiO_2_. The photodegradation reaction rate constant was 0.0281 min^–1^ for NiO/Ag/TiO_2_ and 0.0111 min^–1^ for the TiO_2_ photocatalyst, which is 2.6 times faster under the same condition.

**Table 3:** Reaction Rate Constants and Degradation Efficiencies for Ternary Titania-Supported Silver–Nickel Oxide Nanocomposite (NiO/Ag/TiO_2_) Photocatalysts with Different Doses (0, 0.2, 0.5, and 1 g/L) in MB Aqueous Solution under Visible Light for 60 min

material (g/L)	reaction rate constant (min^–1^)	degradation efficiency (%)
0	0.0027	19.3
0.2	0.0188	56.85
0.5	0.0244	79.71
1	0.0319	93.19

For further characterization, the total organic carbon (TOC) was obtained to measure the carbon amount in the MB aqueous solution before and after adding the catalyst (NiO/Ag/TiO_2_) nanoparticles. The TOC of the solution before adding the catalyst was 85.63 mg/L, and it was 6.07 mg/L after adding NiO/Ag/TiO_2_. The removal of TOC of the MB solution was 92.91% with NiO/Ag/TiO_2_ nanoparticles after 60 min under visible light irradiation, which confirms that the material shows a high photocatalytic activity by degrading the MB solution.

#### 3.3.3 Effect of Annealing

**Figure 9:**
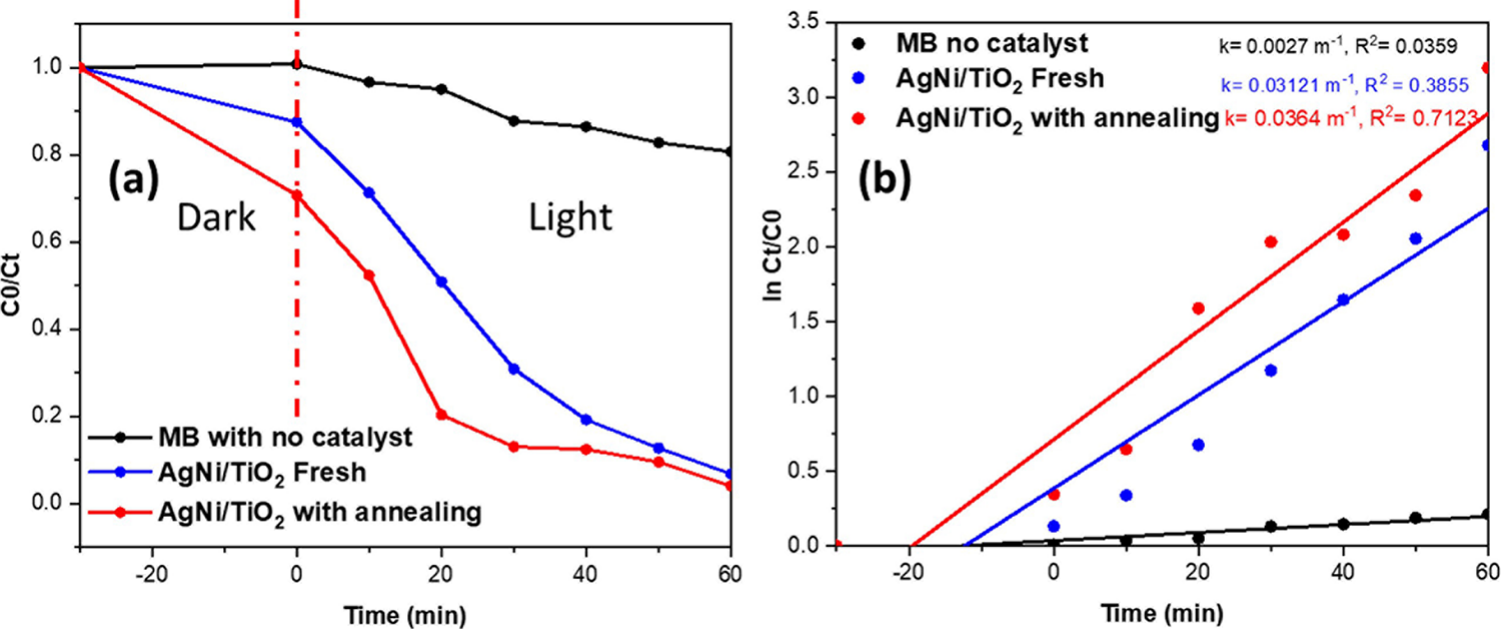
(a) Photocatalytic degradation of MB solution by ternary titania-supported silver–nickel oxide nanocomposite (NiO/Ag/TiO_2_) with annealing (red), without annealing (blue), and with no catalyst (black) under visible light for 60 min. (b) Pseudo-first-order kinetics of the degradation of MB solution.

The annealing treatment can enhance the material’s structural, morphological, electrical, and optical properties.^54^ Recent studies illustrate that annealing will improve the crystalline structure and phase transformation of bare TiO_2_ and Ag/TiO_2_.^55^Figure 9 displays the photocatalytic degradation of MB dye solution by NiO/Ag/TiO_2_ after annealing at 600 °C for 2 h and NiO/Ag/TiO_2_ without annealing. The photocatalytic performance was tested under the same conditions as in all of the experiments in this work. According to Figure 9a, the MB photodegradation by NiO/Ag/TiO_2_ after annealing shows a better decline in the curve of the concentration of MB (C_t_/C_0_) compared to NiO/Ag/TiO_2_ without annealing. Figure 9 indicates the pseudo-first-order kinetics fitted with the degradation data of the MB solution. Figure 9b proves that the NiO/Ag/TiO2 curve with annealing has the fastest reaction rate. Table 4 summarizes the reaction rates and the degradation efficiencies, which report that the catalyst with annealing performs better in the photodegradation activity. Hence, the heat treatment method was used to optimize the properties of the nanocomposite catalyst, which caused an increase in the surface roughness and crystallinity and reduced the nanocomposite’s band gap, leading to advancement in the performance of the photocatalytic degradation toward MB dye solution.^56^ The annealing effect was investigated further and found to have a direct effect on the catalyst performance. Figure 2d illustrates the XRD pattern for NiO/Ag/TiO_2_ before and after annealing, which confirms that the crystallinity of TiO_2_ improved after annealing at 600 °C by increasing the intensity of the peak (110). Also, the observed peaks become sharper after annealing, which enhances the crystallinity of TiO_2_ (rutile) after the thermal treatment. In addition, a Tauc plot of NiO/Ag/TiO_2_ has been employed after annealing, representing a reduction in the band gap reaching 2.2 eV, as shown in Figure S2. Furthermore, annealing leads to the segregation of Ag atoms on the surface of the catalyst due to the lower surface energy of Ag and the reduction of stress due to the large Ag atoms. This behavior was observed in the Ag/TiO_2_ nanocomposite, which can be attributed to the high cohesive energy of Ag nanoparticles and the low interaction energy of silver with NiO and TiO_2_, which increase the mobility of Ag atom on the nanocomposite surface and the agglomeration of Ag nanoparticles.^57^

**Table 4:** Reaction Rate Constants and Degradation Efficiencies for Ternary Titania-Supported Silver–Nickel Oxide Nanocomposite (NiO/Ag/TiO_2_) with and without Annealing and MB Aqueous Solution without Any Catalyst as Control under Visible Light for 60 min

material	reaction rate constant (min^–1^)	degradation efficiency (%)
no catalyst	0.00269	19.3
no annealing	0.03121	93.15
with annealing	0.03635	95.92

#### 3.3.4 Recyclability

**Figure 10:**
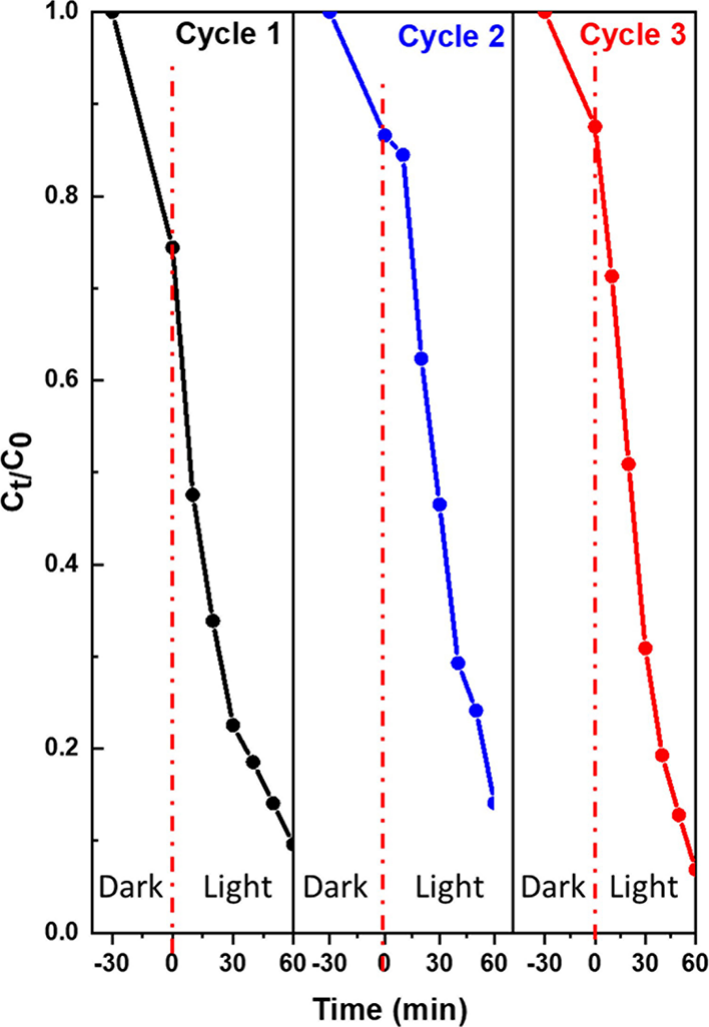
Stability and recyclability evaluation of ternary titania-supported silver–nickel oxide nanocomposite (NiO/Ag/TiO_2_) for photocatalytic degradation of aqueous MB solution under visible light irradiation.

To investigate the recyclability and photostability of the photocatalyst, the photocatalytic activity of NiO/Ag/TiO_2_ (1 g/L) was measured three times under the same conditions on the same photocatalyst after capturing and drying the catalyst. As reported in Figure 10, there are no significant differences in the photocatalyst performance, even after three cycles. This experiment indicates that the NiO/Ag/TiO_2_ photocatalyst has excellent stability and recyclability and can be used efficiently in organic waste degradation.

#### 3.3.5 Photodegradation Mechanism on NiO/Ag/TiO_2_ Nanoparticles under Visible Light Irradiation

The photodegradation mechanism depends on the decomposition of the organic pollutant to toxic compounds such as CO_2_, nitrate, ammonium, and sulfate ions by OH^·^ or O_2_^·–^ radicals, as shown in the following eqs 4–11.^58^
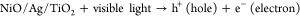
4
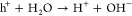
5
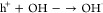
6
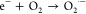
7
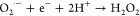
8
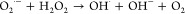
9
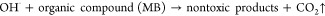
10
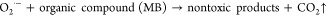
11

**Figure 11:**
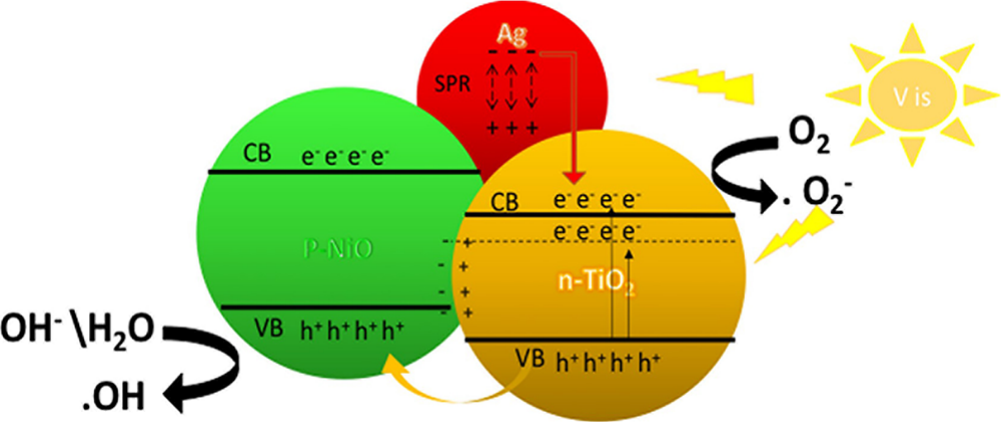
MB photodegradation reaction mechanism on ternary titania-supported silver–nickel oxide nanocomposite (NiO/Ag/TiO_2_) under visible light irradiation.

This approach combined the metal oxide NiO with the Ag metal and the semiconductor metal oxide TiO_2_. The mechanism of producing radicals by electron–hole generation is explained in Figure 11. Due to the specific combination of NiO/Ag/TiO_2_, the metal and metal-oxide interfaces are formed, producing a Schottky barrier.^49^ TiO_2_ NPs can relatively excite the electrons either from the valence band (VB) to the conduction band (CB) under visible light or from the VB to the midband gap transitions (i.e., from the CB to Ti^3+^/Ov states), which is located below the CB, thus allowing for faster transfer of the electrons from the VB to CB.^6,59^

In addition, the presence of Ag NPs within the NiO/TiO_2_ composite results in a further redshift as presented in the UV–vis spectra, which enhances the performance of the composite by decreasing the band gap and makes it a more effective photocatalyst under visible light irradiation due to the LSPR.^60^ Therefore, Ag NPs combined with the surfaces of NiO and TiO_2_ act as a photosensitizer and transfer more electrons to the CB of TiO_2_.^39,61^ The Ag NPs may also transfer electrons to the NiO’s CB; therefore, due to the p-n injection between NiO and TiO_2_, these electrons will move to the CB of TiO_2_.^39,61^ The Schottky junction and the built-in electric field result in a downward movement of electrons from NiO to TiO_2_.^59^ Moreover, the photogenerated holes in the VB of TiO_2_ will transfer to the VB of NiO.^59^ This method efficiently prevented electron–hole recombination, allowing more active electrons to be accessed for radical production. Moreover, the comparative investigation highlighted that Ag NPs in the composite would improve the separation efficiency.^39^ The main reason is the formation of Schottky junctions at the Ag–TiO_2_ and Ag–NiO interfaces.^39^ O_2_ can trap the CB electrons on hybrid shells, producing oxygen-free radicals (·O_2_^–^). Meanwhile, the holes in the VB react with H_2_O or OH to form ·OH. As a result, the free radicals degrade the MB dye component into nontoxic products and CO_2_ gas.^6,39,46,59,62^

For further investigation into the mechanism of photodegradation activity, it is necessary to detect the significant active species, such as trapped electrons, trapped holes, ·O^2–^, and ·OH accurately and consistently by using several techniques along with using electron resonance spectroscopy.^62,63^

### 3.4 Photodegradation Activity for Drugs (ASP and PCM)

**Figure 12:**
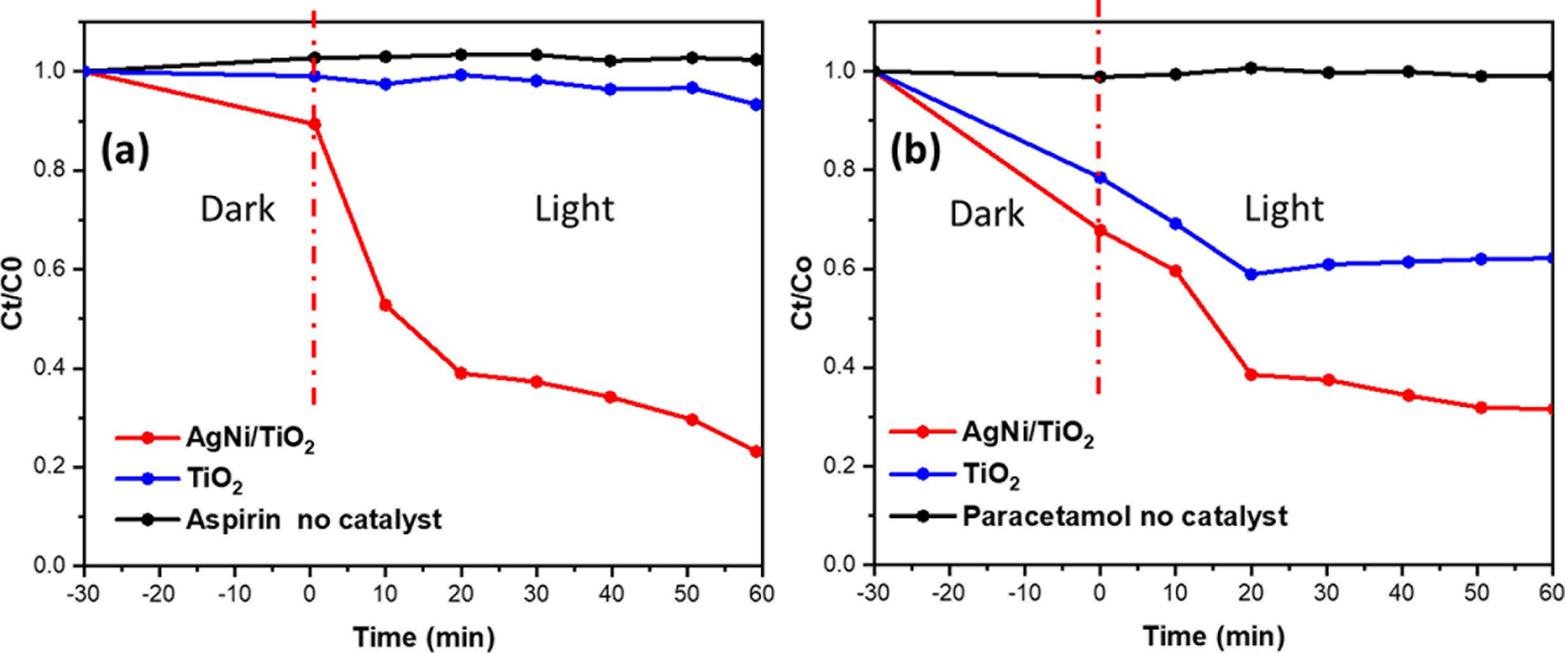
Degradation rate of pharmaceutical drugs by ternary titania-supported silver–nickel oxide nanocomposite (NiO/Ag/TiO_2_) and TiO_2_ photocatalysts. (a) ASP and (b) PCM under visible light for 60 min.

The NiO/Ag/TiO_2_ nanocatalyst was assessed in the dissociation of the ASP and PCM compounds. The ASP and PCM have different functional groups, which offer various linking sites for the photocatalyst. Two experiments were done in the same condition for 60 min for both ASP and PCM with TiO_2_ as a reference and NiO/Ag/TiO_2_ nanocomposite under visible light irradiation. According to Figure 12, ASP and PCM show that NiO/Ag/TiO_2_ has higher photocatalytic activity than bare TiO_2_. Thus, the larger the number of binding sites for the adsorption of medicinal compounds on catalysts, the better the photocatalytic activity. The electron–hole pairs are formed by visible light irradiation, which produces the oxide and hydroxyl radicals from O_2_, and H_2_O is absorbed through the photocatalysts’ surfaces. These radicals oxidize pharmaceutical substances, producing CO_2_ and H_2_O and other byproducts.^2,64^ The degradation of pharmaceutical drugs can be divided into two categories, as determined by the reaction mechanisms: (a) direct charge transfer via the produced composite material photocatalyst to the soluble drug molecules and (b) generated free radicals from the breakdown of water, which then attack the contaminants.^65^ Based on these results, the full degradation of PCM and ASP was not achieved.

To understand the reaction mechanism and the active species for the degradation of ASP and PCM by Ag/NiO/TiO_2_, further analysis has been done for the degradation of ASP and PCM by Ag/NiO/TiO_2_ to confirm the creation of oxidation products (OPs) which are the means responsible for the decomposition of the drugs compound. HPLC of Figures S3 and S4 depict the ASP and PCM degradation and the presence of the OPs, respectively. The retention times for ASP and PCM were around 0.985 and 1.2 min, respectively.

Typically, five OPs have been detected at 1.236, 1.405, 1.655, and 1.893 min during ASP degradation, as shown in Figure S3. The ASP peak reduced drastically as the time increased, which means it was dissociated from other OPs, which are OP1, OP2, OP3, OP4, and OP5. It is significant to mention that the OP1, after 40 min, degraded exponentially and disappeared at 120 min, which means that the OPs will degrade after a while.^66^ To identify the OPs, it is important to understand the pathway of the degradation of ASP, which starts with hydrolyzation in water to form (1) salicylic acid and fumaric acid, (2) acetic acid, or (3) maleic acid, malic acid, and malonic acid. The next step is the oxidation of salicylic acid to form 1,2-dihydroxybenzene, which eventually oxidizes to form hydroquinone. Moreover, part of the salicylic acid is oxidized to form 2,3-dihydroxybenzoic acid. Since the hydroquinone hydroxylated to benzoquinone is unstable in an aqueous solution, it will undergo ring-opening followed by oxidation to muonic acid.^67^ The hydroxyl radical addition to the double bond of muonic acid produced maleic and oxalic acid. The oxidation of maleic/fumaric acid produces malic acid, which is a malonic acid precursor. Then, the malonic acid produces acetic acid and carbon dioxide.^68^ Oxalic acid oxidation to CO_2_ occurs via the formation of formic acid. This mechanism fits well with the photo-Kolbe reaction pathway, where the adsorbed CH_3_COOH dissociated to CH_3_COO^–^ species, which reacted with photogenerated holes to form CH_3_ radicals and CO_2_.^69^ The CH_3_ radicals then react with H_2_O to form CH_4_ or C_2_H_6_, and CH_3_ radicals also react with OH radicals to generate CH_3_OH. The photogenerated holes attack the produced CH_3_OH to produce HCHO, which is oxidized by the OH radicals, and the photogenerated holes produce HCOOH.^70^ Finally, HCOOH undergoes a decarboxylation reaction through the photo-Kolbe reaction to produce CO_2_.^70^

In the case of PCM, Figure S4 shows the dissociation of the PCM compounds to other OPs. Three main OPs have been found at 1.856, 1.67, and 1.919 min for OP1, OP2, and OP3, respectively.^71^ These results are mainly attributed to forming organic intermediates that remain within the reaction, such as 4-aminophenol, due to successive hydroxylation and oxygenation, which eventually degrades into hydroquinone.^72^ This process proceeds by para-substitution of the nitrogenous group by a hydroxyl group, followed by subsequent removal of the functional group containing nitrogen. Successive oxygenation in the reaction medium generates 1,4-benzoquinone through the oxidation of 1,4-dihydroxybenzene or hydroquinone.^73^ The aromatic ring intermediate carbonyl organic compound might be fragmented into species such as carboxylic acids, esters, or compounds containing the carbonyl group (C=O), as well as nitrates and nitrites as a result of the oxidation of the nitrogenous group’s presence in the reaction.^74^

## 4 Conclusions

In summary, the NiO/Ag/TiO_2_ ternary heterojunction nanocomposite was successfully synthesized by depositing and distributing the Ag and NiO nanoparticles uniformly in spherical shapes with sizes <10 nm on the surface of TiO_2_ with a size range of 150–200 nm using a coprecipitation method. XRD, TEM, TEM-EDS mapping, HRTEM, and XPS were employed to characterize the prepared material. The metallic Ag and NiO are well dispersed on the tetragonal rutile TiO_2_ with no other phase. The higher porosity and the reduction in the band gap led to the redshift in the UV-absorption spectra and the high production of radicals compared to the other composites (Ag/TiO_2_, NiO/TiO_2_, and bare TiO_2_) and are responsible for the photocatalytic activity of NiO/Ag/TiO_2_. NiO/Ag/TiO_2_ showed excellent photocatalytic efficiencies for organic compound degradation, such as pharmaceutical wastes (i.e., ASP and PCM) and dyes such as MB with high stability and recyclability. Ag NPs play a crucial role in enhancing the catalytic activity and facilitating the electron transfer, suppressing the electron–hole recombination by acting as a photosensitizer and a photocatalyst by transferring additional electrons to the CB of TiO_2_. LSPR, the intimate contact of TiO_2_, NiO, and Ag through the formation of Schottky junctions at the Ag–TiO_2_ and Ag–NiO interfaces, and the formation of a p–n junction between NiO and TiO_2_ within the nanocomposite structure all play a crucial role in the photocatalytic activity by reducing the band gap energy, shifting the absorption to the visible light region, and enhancing the electron–hole pair separation efficiency. This research shows that the photocatalyst is very efficient in degrading organic compounds with suitable efficiency. Further studies are required to evaluate the toxicity and inhibition of microorganisms (i.e., bacteria and viruses) of the ternary NiO/Ag/TiO_2_ nanocomposite.

## Supporting Information Available

Schematic of the ternary NiO/Ag/TiO_2_ heterojunction nanocomposite synthesis procedure; XPS survey scan showing the chemical components of the composite; band gaps of the composites using the Tauc plot calculation of the NiO/Ag/TiO_2_ nanocomposite after annealing; representative HPLC of ASP compound degradation by Ag/TiO_2_/NiO nanoparticles; and representative HPLC of PCM compound degradation by Ag/TiO2/NiO nanoparticles (PDF)
