# Supplementary material for: Visible Light Photocatalytic Degradation of Methylene Blue Dye and Pharmaceutical Wastes over Ternary NiO/Ag/TiO2 Heterojunction
Source: ACS Omega. 2023 Oct 20;8(43):40063–77. doi: 10.1021/acsomega.3c01766 (PMC10620881; doi:10.1021/acsomega.3c01766)
Supplement: Supplementary file 1 — ao3c01766_si_001.pdf [file ao3c01766_si_001.pdf]

Supplementary materials

Visible Light Photocatalytic Degradation of Methylene Blue (MB) Dye and Pharmaceutical Wastes Over Ternary $NiO/Ag/TiO_2$ Heterojunction

Widad Mohammed[1], Maha Matalkeh[2], Rola Mohammad Al Soubaihi[3], Ahmed Elzatahry[1], Khaled M Saoud[2*]

[1] Material Science and Technology Program, College of Arts and Sciences, Qatar University, 2713, Doha, Qatar.

[2] Liberal Arts and Science, Virginia Commonwealth University School of Arts in Qatar, PO Box 8095, Doha, Qatar.

[3] Functional NanoMaterials Group, Department of Applied Physics, School of Engineering Sciences, KTH Royal Institute of Technology, Hannes Alfvéns väg 12, 11419 Stockholm, Sweden

Content of the material Supplied as Supporting Information FIGURES' CAPTIONS

Figure S1: Schematic of the ternary NiO/Ag/TiO2 heterojunction nanocomposite synthesis procedure.

Figure S2: XPS survey scan showing the chemical components of the composite.

Figure S3: Band gaps of the composites using the Tauc plot calculation of $NiO/Ag/TiO_2$ nanocomposite after annealing.

Figure S4: Representative HPLC of ASP compound degradation by $Ag/TiO_2/NiO$ nanoparticles a) ASP without catalyst b) after 40 min with catalyst c) at 80 min d) at 120 min.

Figure S5: Representative HPLC of PCM compound degradation by Ag/TiO2/NiO nanoparticles a) PCM without catalyst b) after 40 min with catalyst c) at 80 min d) at 120 min.

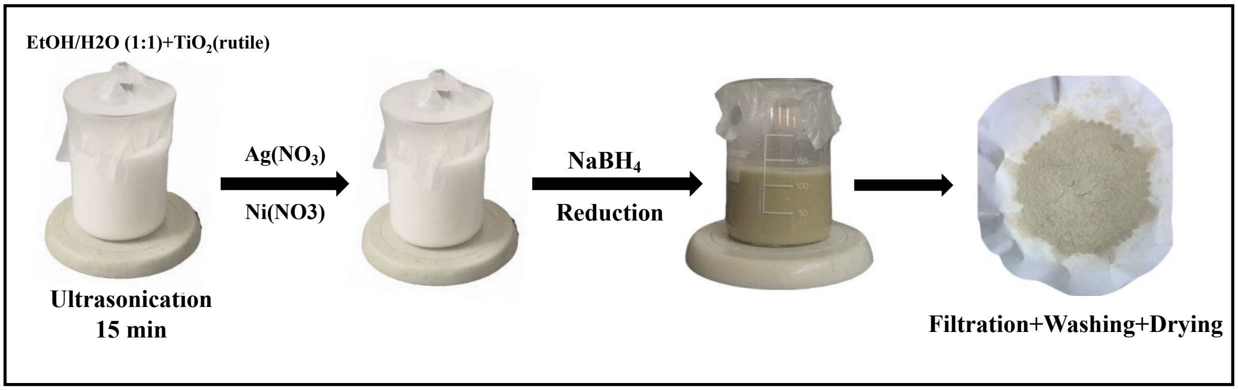

Figure S2: Schematic of the ternary NiO/Ag/TiO2 heterojunction nanocomposite synthesis procedure.

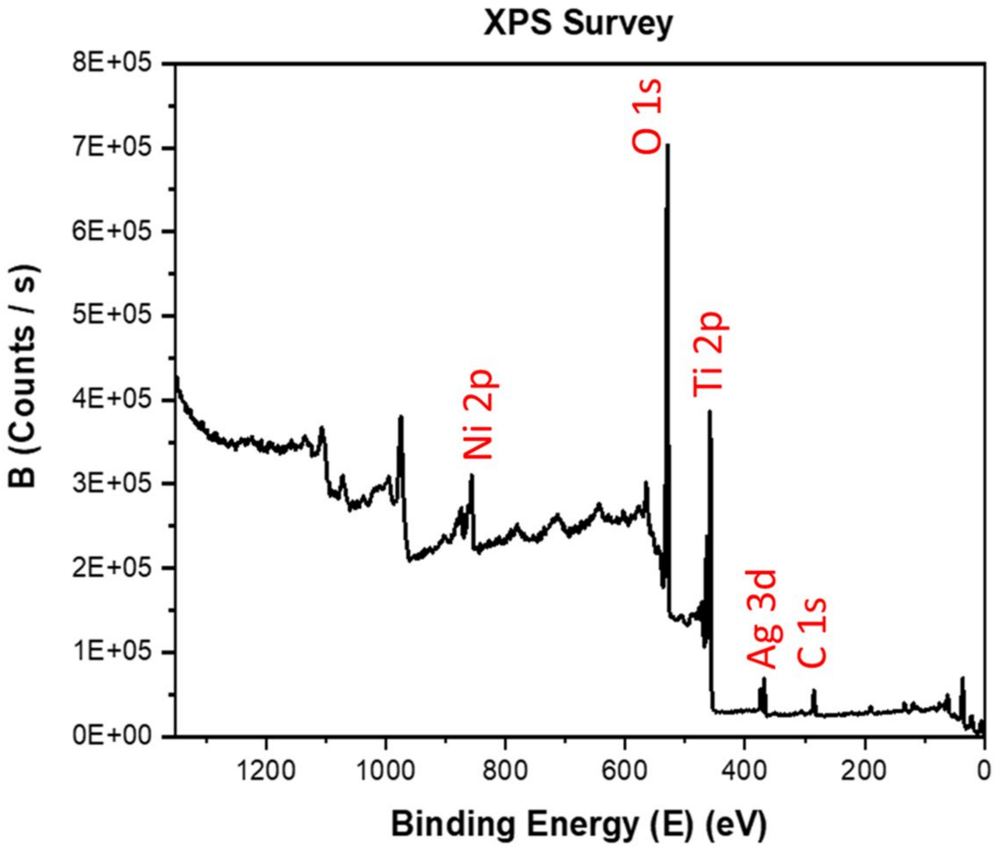

Figure S2: XPS survey scan showing the chemical components of the NiO/Ag/TiO_2 nanocomposite.

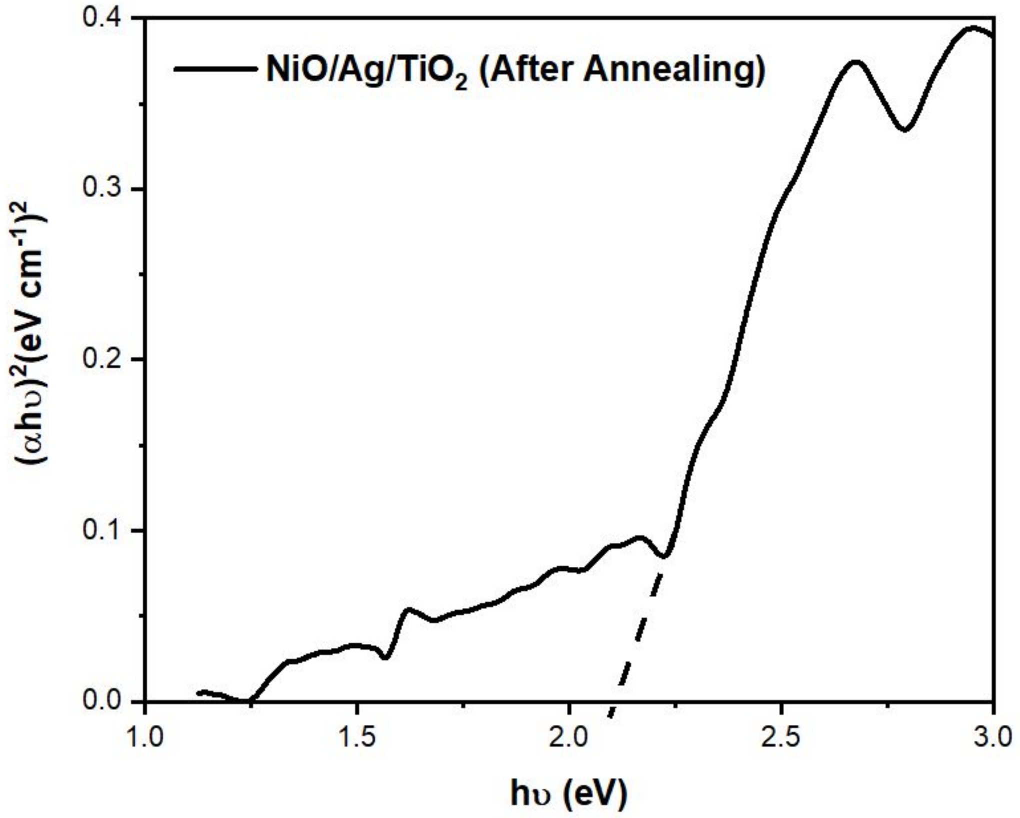

Figure S3: Band gaps of the composites using the Tauc plot calculation of NiO/Ag/TiO_2 nanocomposite after annealing.

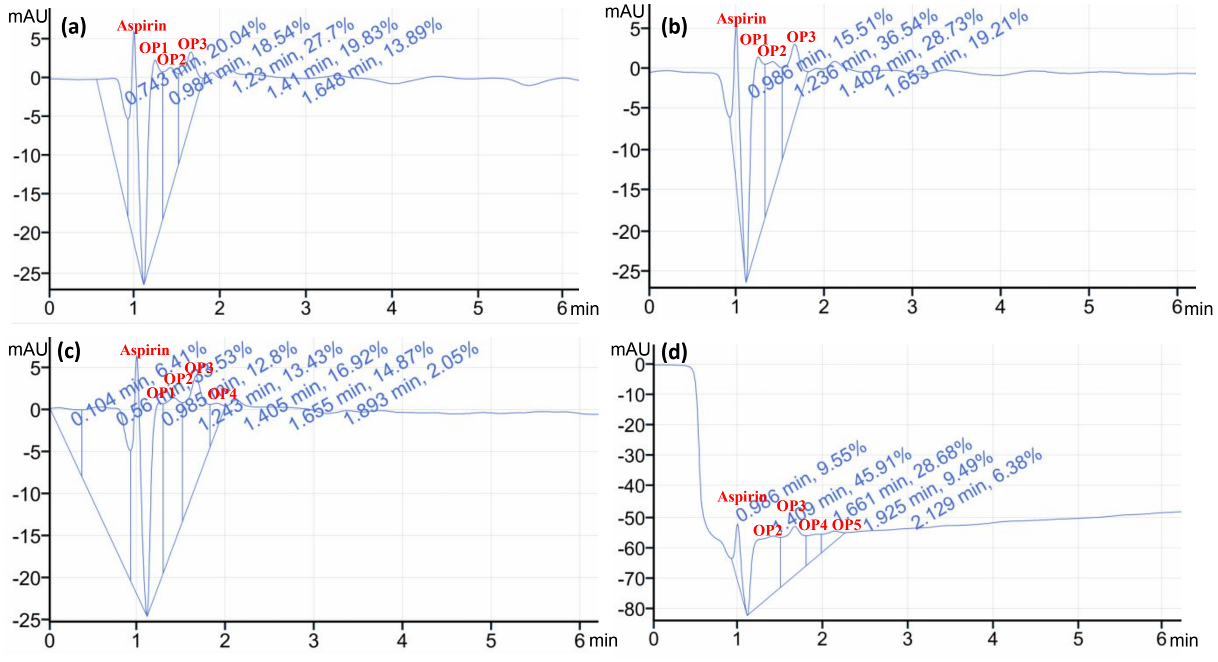

Figure S4: Representative HPLC of ASP compound degradation by Ag/TiO_2/NiO nanoparticles a) ASP without catalyst b) after 40 min with catalyst c) at 80 min d) at 120 min.

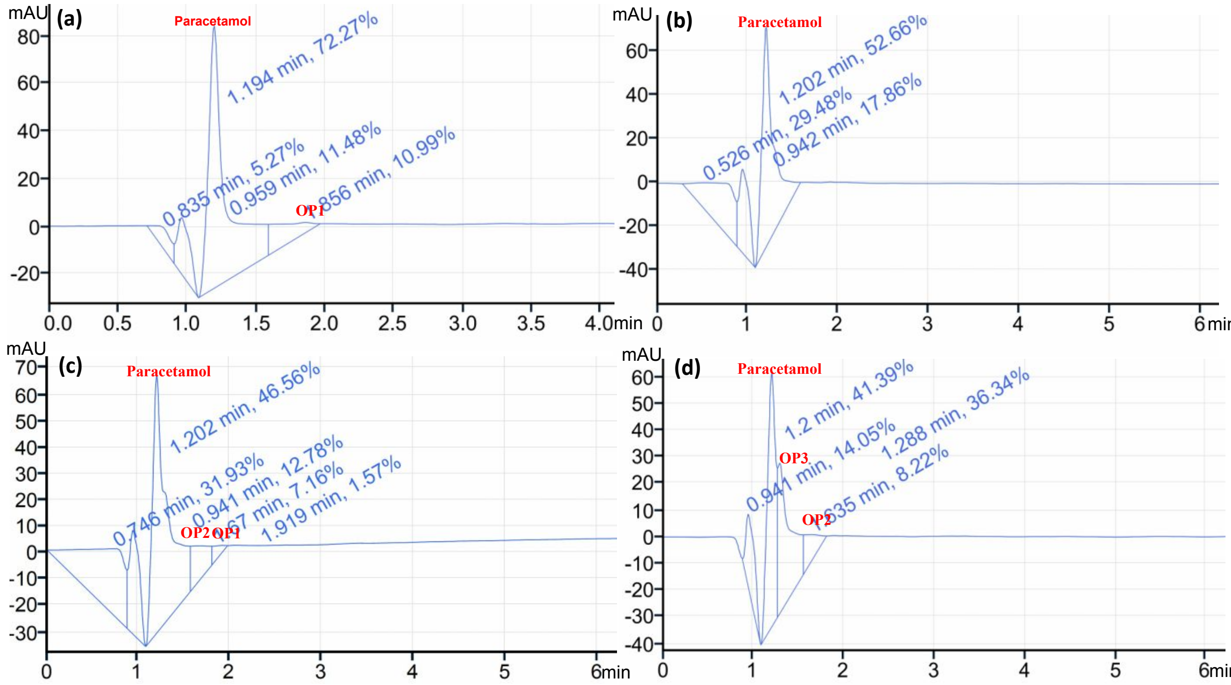

Figure S5: Representative HPLC of PCM compound degradation by Ag/TiO2/NiO nanoparticles a) PCM without catalyst b) after 40 min with catalyst c) at 80 min d) at 120 min.
